# The Development and Initial End-Point User Feedback of a 3D-Printed Adult Proximal Tibia IO Simulator

**DOI:** 10.7759/cureus.25481

**Published:** 2022-05-30

**Authors:** Mithusa Sivanathan, Julia Micallef, Krystina M Clarke, Bruno Gino, Shitji Joshi, Sandy Abdo, Dania Buttu, Marvin Mnaymneh, Samyah Siraj, Andrei Torres, Gordon Brock, Dale Button, Carla Pereira, Adam Dubrowski

**Affiliations:** 1 Health Sciences, Ontario Tech University, Oshawa, CAN; 2 Emergency Medicine, Faculty of Medicine, Memorial University of Newfoundland, St. John's, CAN; 3 Emergency Medicine, Madrecor Hospital, Uberlândia, BRA; 4 Engineering and Applied Science, Ontario Tech University, Oshawa, CAN; 5 Education, Ontario Tech University, Oshawa, CAN; 6 Computer Science, Ontario Tech University, Oshawa, CAN; 7 Family Practice, Centre De Sante Temiscaming, Temiscaming, CAN; 8 Paramedicine, Durham College, Oshawa, CAN; 9 Allergy and Immunology, Uberlândia Medical Centre (UMC), Uberlândia, BRA

**Keywords:** psychomotor skills, io, emergency medicine, additive manufacturing, three-dimensional (3d) printing, simulation-based medical education, intraosseous infusion, simulator, training, 3d-printing

## Abstract

Intraosseous infusion (IO) remains an underutilized technique for obtaining vascular access in adults, despite its potentially life-saving benefits in trauma patients. In rural and remote areas, shortage of training equipment and human capacity (i.e., simulators) are the main contributors to the shortage of local training courses aiming at the development and maintenance of IO skills. Specifically, current training equipment options available for trainees include commercially available simulators, which are often expensive, or animal tissues, which lack human anatomical features that are necessary for optimal learning and pose logistical and ethical issues related to practice on live animals. Three-dimensional (3D) printing provides the means to create cost-effective, anatomically correct simulators for practicing IO where existing simulators may be difficult to access, especially in remote areas. This technical report aims to describe the development of maxSIMIO, a 3D-printed adult proximal tibia IO simulator, and present feedback on the design features from a clinical co-design team consisting of 18 end-point users.

Overall, the majority of the feedback was positive and highlighted that the maxSIMIO simulator was helpful for learning and developing the IO technique. The majority of the clinical team responders also agreed that the simulator was more anatomically accurate compared to other simulators they have used in the past. Finally, the survey results indicated that on average, the simulator is acceptable as a training tool. Notable suggestions for improvement included increasing the stability of the individual parts of the model (such as tightening the skin and securing the bones), enhancing the anatomical accuracy of the experience (such as adding a fibula), making the bones harder, increasing the size of the patella, making it more modular (to minimize costs related to maintenance), and improving the anatomical positioning of the knee joint (i.e., slightly bent in the knee joint). In summary, the clinical team, located in rural and remote areas in Canada, found the 3D-printed simulator to be a functional tool for practicing the intraosseous technique. The outcome of this report supports the use of this cost-effective simulator for simulation-based medical education for remote and rural areas anywhere in the world.

## Introduction

Simulation-based medical education (SBME) is a rapidly evolving discipline that allows healthcare providers in practice and training to learn and sustain clinical processes without endangering patients [1]. Three-dimensional (3D) printing has been introduced into health professions education as a low-cost alternative to costly simulators for learning procedural skills [2]. Emergency medicine, particularly in remote areas, can benefit from SBME because it allows students and professionals to practice rare but possibly life-saving skills in a controlled and safe environment. An example of high acuity, low occurrence skill is intraosseous (IO) infusion [3,4].

The IO procedure injects fluid directly into the bone marrow, where it is quickly absorbed by the body. When peripheral intravenous (IV) access is difficult or not possible, IO access provides a viable route to vascular circulation [5]. It is also superior to other alternative routes, such as central venous catheterization (CVC), in terms of success rate and access time [6,7]. As a result, IO is most commonly utilized in emergencies, where quick vascular access is critical for patients who are suffering from shock, serious trauma, cardiac arrest, or other situations needing urgent fluid administration. Despite recommendations from organizations such as the American Heart Association Guidelines for Advanced Cardiovascular Life Support and the European Resuscitation Council Guidelines [7,8], it is frequently underutilized in the adult population, being used only as a fourth option after multiple failed attempts at IV and CVC [5].

In rural and remote areas, a shortage of training equipment (i.e., simulators) and human capacity (i.e., medical educators) are the main contributors to the shortage of local training courses aiming at the development and maintenance of IO skills [9]. Specifically, current training equipment options available for trainees include commercially available simulators, which are often expensive, or animal tissues, which lack human anatomical features necessary for optimal learning and pose logistical and ethical issues related to practicing on animals. 3D printing allows for the creation of cost-effective, anatomically realistic simulators for practicing IO in situations where existing simulators are difficult to access [3].

The purpose of this technical report is to describe the development and initial end-point user feedback of maxSIMIO, a 3D-printed adult proximal tibia IO simulator that could offer a more accessible means. Using a modified context, input, process, and product structure, we aim to provide a recipe for other users to optimize the applicability of the developed simulator to various learning environments, educational contexts, inputs, processes, and expected outcomes [10].

## Technical report

Context

maxSIMIO was made with the intent to train medical students and advanced care paramedics who have limited experience with the IO technique in a remote setting. Three collaborative groups with complementary areas of expertise collaborated to design this simulator: technical designers and graduate students from maxSIMhealth laboratory, a research laboratory located at Ontario Tech University in Oshawa, Ontario, Canada (hereafter referred to as the development team); a group of practicing rural doctors who are members of the Society for Rural Physicians of Canada; and advanced care paramedics at the Durham Region Health Department, Region of Durham Paramedic Services, The Central East Prehospital Care Program, Lakeridge Health, and Durham College (hereafter referred to as the clinical team).

Co-design and initial feedback from the clinical team were gathered iteratively during multiple hands-on agile design sessions to improve the simulator. The feedback on the final design was provided by a group of remote and rural doctors during the 29^th^ Annual Rural and Remote Medicine Course conference on April 22, 2022, hosted by the Society of Rural Physicians of Canada in Ottawa, Ontario, Canada. 

Inputs and design process

Architecture of maxSIMIO

The development and clinical teams brainstormed design ideas for the simulator initially. The designs of the simulator were created by the development team using publicly available medical images or digital models licensed by Creative Commons (CC BY-NC-SA 4.0, Creative Commons, Mountain View, CA, USA) and finalized using subsequent feedback from the clinical team. The designs were saved as stereolithography (STL) files in SolidWorks (Dassault Systèmes SolidWorks Corporation, Waltham, MA) and then prepared for 3D printing using Ultimaker Cura (Ultimaker B.V., Utrecht, Netherlands) 3D slicing software. The 3D-rendered files were then transferred to an Ultimaker S5 3D printer (Ultimaker B.V., Utrecht, Netherlands) using a secure digital (SD) card.

**Figure 1:**
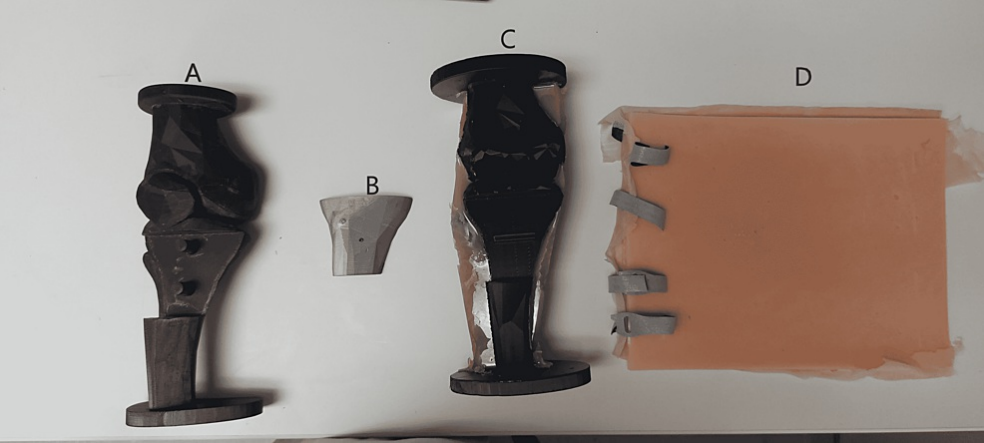
Components of maxSIMIO Straight Leg Model: A) Base, B) replaceable tibial cartridge, C) bone and muscle tissue, and D) skin attachment.

There are two versions of the maxSIMIO simulator: the first version was developed before the workshop and used to gather feedback, and the second version was developed after receiving feedback from the workshop. The first model (straight leg) has the knee orientated in its fully extended position resulting in a straight leg. This version consists of four parts (Figure 1); A) the base, B) the replaceable cartridge, C) the muscle, D) and the skin. All of the 3D printed parts were printed using polylactic acid (PLA), with polyvinyl alcohol (PVA) being used to print the supporting structures. These parts were printed using the Ultimaker S5 3D printers. All of the silicone parts were created by mixing Dragon Skin™ 10 NV (Smooth-On, Macungie, PA) with a small amount of Silc-Pig™ silicone pigment (Smooth-On, Macungie, PA).

The design for the straight leg model was created by manipulating surface scans of the femur, tibia, fibula, and patella bones obtained through a Creative Commons license (CC BY-NC-SA 4.0, Creative Commons, Mountain View, CA, USA). These files were adjusted to the correct orientation using the SolidWorks Assembly feature. Then, by using a series of plane cuts, the model was cut [into/down] to a more manageable size. All of the solids were then merged into one single solid using the combine feature. To obtain the replaceable cartridge, three plane cuts were made on the main model with the merge setting turned off, which resulted in two solids. The cartridge was then exported as an STL file. The remaining solid was then manipulated further by sketching and extruding thin cylinders at the ends to cap off the model. This remaining solid body of the bones was then also exported as an STL file. These STL files were then finalized in Autodesk Meshmixer (Autodesk, Inc., San Rafael, CA), using its hollow feature to make the cartridge and bones hollow. In addition to these parts, a mold was required for the manufacturing of the skin. This mold was created in SolidWorks by sketching two rectangles and extruding them to create a simple box shape. All of the digital files developed for this project are publicly available on GitHub and can be found at the following link: https://github.com/maxSIMhealth/maxSIMIOv1.0.

To make the muscle layer of the model, a mold was created out of recycled cylindrical storage tins. This served as an affordable and environmentally friendly method to make the muscle since the containers were being reused for this purpose. The muscle was created by cutting containers in half longitudinally. Then, the 3D printed model of the bones was placed inside the containers, and the ends of the model were taped together with the mold using duct tape. Lastly, Dragon Skin™ 10 mixed with red Silc-Pig™ silicone pigment was poured into the mold, and once it cured after approximately 75 minutes, the mold was removed, and the muscle layer was complete.

To make the skin layer, a piece of power mesh fabric was placed into the skin mold, improving the strength of the skin layer. Then, skin-coloured Silc-Pig™ silicone pigment was mixed into the Dragon Skin™ 10 NV silicone and poured into the skin mold. After curing for approximately 75 minutes, five vertical slits were cut into the ends of the skin. The replacement cartridge was installed on the model, and the skin was stretched around to enclose it. Lastly, straps made from Velcro were used to secure the skin and keep it in place, which provides an easy method to replace the skin and cartilage.

**Figure 2:**
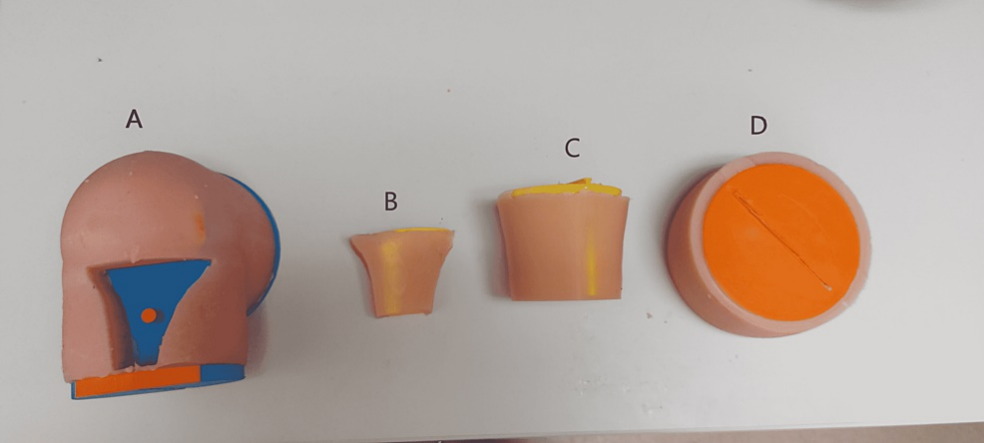
Components of maxSIMIO Bent Knee Model: A) Base, B) replaceable tibial cartridge, C) lower leg (tibia and fibula), and D) upper leg (femur).

In the second model of the maxSIMIO simulator, feedback was used to improve the first model, resulting in a larger bent-knee design. The new model consisted of four parts (Figure 2), A) the base, B) the replaceable cartridge, C) the lower leg, and D) the upper leg. This design was made modular for portability as well as anatomical accuracy. Similar to the straight leg model, all 3D-printed components were created using PLA filament for the body and PVA filament for the more complex supports. These parts were printed using the Ultimaker S5 printers. All of the silicone components were made by mixing Dragon Skin™ 10 NV with a small amount of Silc-Pig™ silicone pigment.

This design was derived from the same models of the bone as the straight leg model; however, they were arranged so that the knee would be in a 90-degree position. This was done using the SolidWorks assembly feature. Next, a freehand sketch was created around the bones using the spline feature to create the shape of a leg. This sketch was then extruded around the bones using the revolve feature to create two solids, the bones and the leg. Next, the hollow feature was used to hollow out the leg solid, then followed by a series of plane cuts which resulted in splitting up both solids at the femur, knee joint, and lower tibia. A disk was then extruded at the location of the plane cuts to provide an interface for the three components to attach to. These components were then exported as STL files, sliced in Ultimaker Cura, then 3D-printed.

To attach the skin to the model, skin-coloured Silc-Pig™ silicone pigment was mixed into the Dragon Skin™ 10 NV silicone and poured into the different molds that already contained the 3D-printed bones. Once the silicone had cured after approximately 75 minutes, the four separate parts were removed from their molds and attached. This method resulted in an anatomically correct human leg model.

Materials for the IO Station

**Figure 3:**
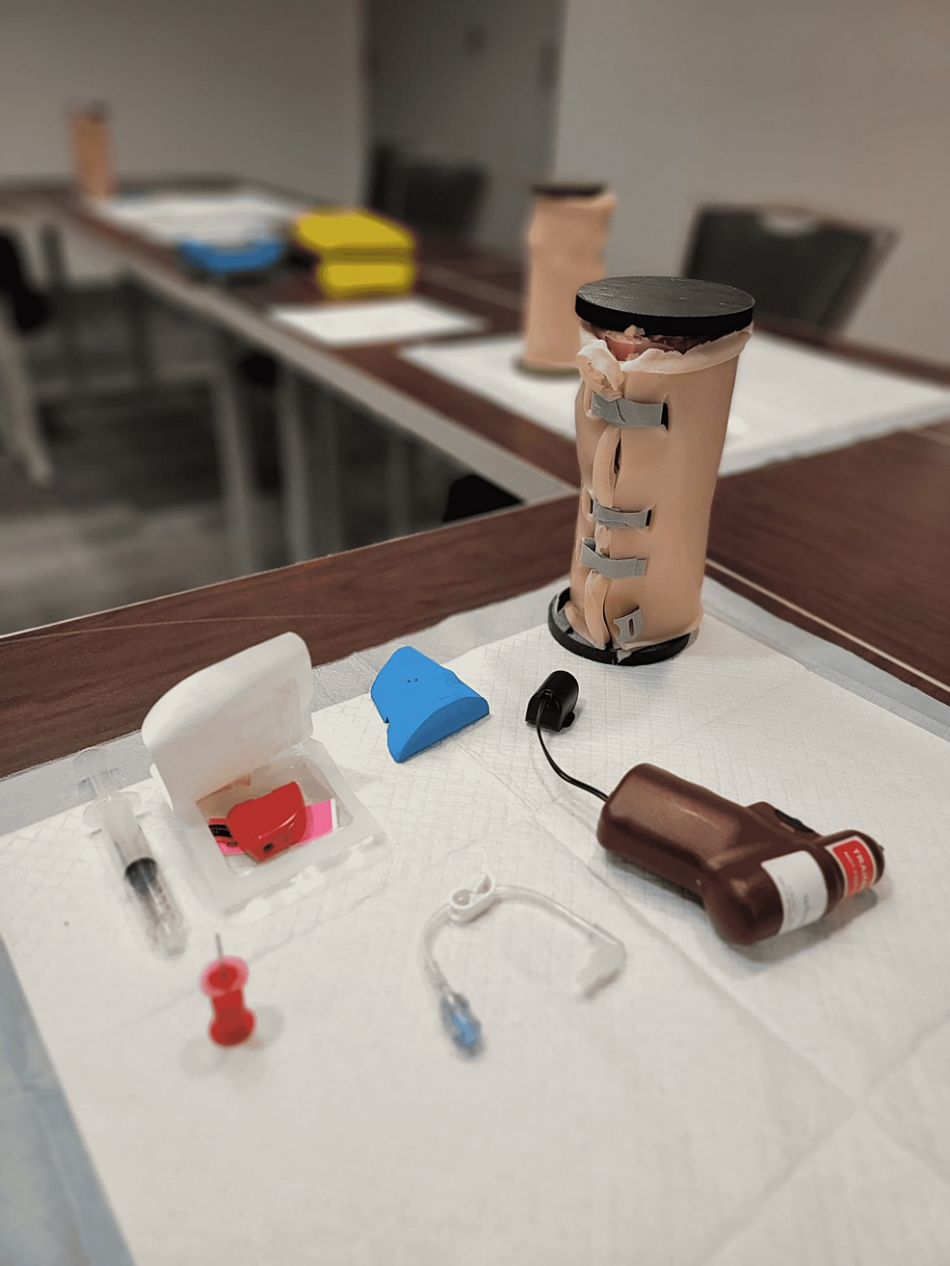
IO station equipment setup showing the maxSIMIO, an Arrow® EZ-IO® Power Driver, and an EZ-IO® Needle Set.

During the final co-design feedback loop, the rural and remote doctors and trainees were able to attempt IO insertion at a training station. The equipment provided included: the maxSIMIO simulator, an Arrow® EZ-IO® Power Driver (Teleflex Medical Research Triangle Park, NC, USA), and an EZ-IO® Needle Set (Figure 3). 

**Figure 4:**
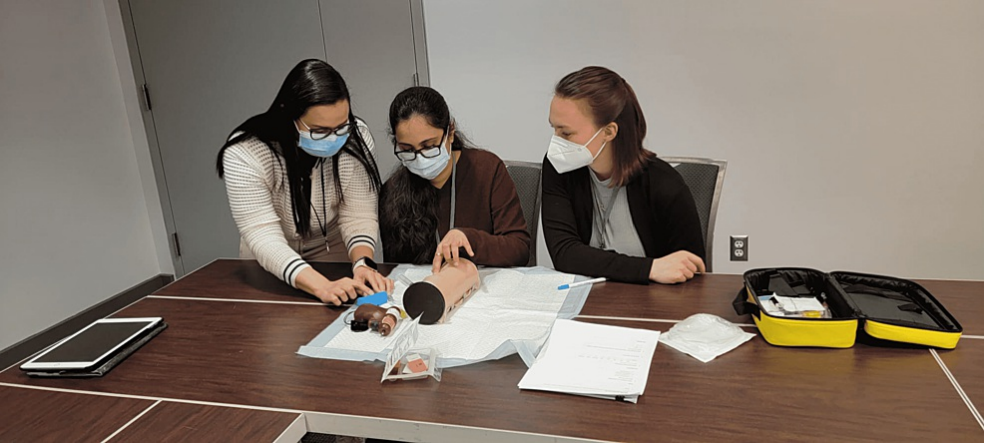
Instructor training participants on the IO technique on maxSIMIO using the Arrow® EZ-IO® Power Driver and EZ-IO® Needle Set.

During the session, the doctors and trainees were first shown a two-minute instructional video on the IO technique performed by a medical professional (video can be found at this link: https://github.com/maxSIMhealth/maxSIMIOv1.0), and, a live instructor as well as members of the development team were present to provide additional instructions and consultations about the technical aspects of the simulator (Figure 4). 

The videos and live instructions highlighted appropriate landmarking, needle insertion, and the attachment of a catheter hub. At the end of the session, the doctors and trainees were asked to complete a survey that assessed maxSIMIO using Likert-type and open-ended questions (Table 1). The survey was developed based on the Michigan Standard Simulation Experience Scale, which allowed the clinical team to express their a) perceptions related to maxSIMIO’s representations of the anatomical features, b) maxSIMIO’s potential to serve as an educational tool, and c) suggest improvements to the maxSIMIO [11]. This co-development session was four hours in length and concluded with a face-to-face debrief.

**Table 1:** Questions of the model assessment survey.

Demographics						
Question Number	Question					
3	What is your level of training?					
4	What is your specialty?					
5	Approximately how many times have you performed the procedure in scope?					
Self-efficacy						
Question Number	Question					
6	The model helped improve my KNOWLEDGE on the procedure in scope					
7	The model helped improve my CONFIDENCE in performing the procedure in scope					
8	The model helped improve my ABILITY in performing the procedure in scope					
9	The model helped improve my ability in performing the procedure in scope INDEPENDENTLY					
10	Comments/suggestions regarding the model that may improve your self-efficacy?					
Fidelity						
Question Number	Question					
11	The model used has anatomically accurate characteristics/features					
12	Did you experience any difficulties or discomfort while using the model?					
13	Comments/suggestions regarding the model to improve the fidelity?					
Educational Value						
Question Number	Question					
14	The model is a good training tool for KNOWLEDGE in the procedure in scope					
15	The model is a good training tool for SKILLS in the procedure in scope					
16	Comments/suggestions regarding the model that may improve its educational value?					
Teaching Quality						
Question Number	Question					
17	The instructor was knowledgeable about the procedure in scope					
18	Instructor was able to convey material on the procedure in scope in a way that was understandable to me					
19	The learning materials/resources provided improved my understanding of the procedure in scope					
20	Comments/suggestions regarding the model that may improve teaching quality?					
Self-efficacy						
Question Number	Question					
21	Overall, the model was a helpful training tool for the procedure in scope					
22	For the evaluation of the model...					
23	What specific changes would you suggest to improve your learning experience?					
24	Other than the model you tried today, have you used another model in the past?					
25	If you have previously used another model, how effective was it in increasing your confidence in performing the procedure in scope?					
26	If you have used another model before, how does this model you tried today compare to the previous model?					

Products/outcomes

Costs

The breakdown of all costs related to the manufacturing of maxSIMIO is shown in Table 2. Based on the production cost of a single maxSIMIO, all cost estimates are in Canadian dollars (CAD), including local taxes.

**Table 2:** Cost breakdown of the materials (in CAD) needed to produce maxSIMIO.

Material	Straight Leg Model (g)	Straight Leg Model Molds (g)	Cost of Straight Leg Model (CAD)	Bent Leg Model (g)	Bent Leg Model Molds (g)	Cost of Bent Leg Model (CAD)
PLA	119	138	6.42	418.6	439	21.43
PVA	106.7	0	21.32	152.7	0	30.52
Dragon Skin™ 10 NV	685	0	31.08	3230.4	0	146.58
TOTAL COST	-	-	58.82	-	-	198.53

User Feedback 

Eighteen end-point users, part of the clinical team, completed the survey, and the results are presented in Table 3. Overall, the majority of the feedback was positive and highlighted that the maxSIMIO simulator helped obtain knowledge and skills on the IO technique. The majority of the clinical team responders also agreed that the simulator was more anatomically accurate compared to other models that they have used in the past. Finally, the survey results indicated, on average, that the simulator is acceptable as an educational training tool.

**Table 3:** Ratings for each of the model assessment questions.

Demographics									
Question Number	Options								Total
	M1	M2	M3	M4	PGY	Resident	Fellow	Attending	
3	3	4		6		2		3	18
	Comments								
4	- IM (S1)		- Family med (S3)		- Family medicine (S4)		- matched FM (S5)		
	- Family medicine (S6)		- FM (S9)		- P.... practice (S11)		- Family medicine (S12)		
	- Student (S14)		- FM (S16)		- Medical Student (S17)		- FM - ESS (S18)		
	Never	1-5	6+						
5	9	7	1						17
Self-efficacy									
Question Number	Scale 1 (strongly disagree) to 5 (strongly agree)					Total	Average Response	Standard Deviation	
	1	2	3	4	5				
6	0	1	2	4	11	18	4.39	0.92	
7	0	0	2	7	9	18	4.39	0.70	
8	0	1	0	7	10	18	4.44	0.78	
9	0	0	3	10	5	18	4.11	0.68	
Fidelity									
Question Number	Scale 1 (strongly disagree) to 5 (strongly agree)					Total	Average Response	Standard Deviation	
	1	2	3	4	5				
11	0	0	5	9	3	17	3.88	0.70	
Educational Value									
Question Number	Scale 1 (strongly disagree) to 5 (strongly agree)					Total	Average Response	Standard Deviation	
	1	2	3	4	5				
14	0	0	1	4	13	18	4.67	0.59	
15	0	0	1	4	13	18	4.67	0.59	
Teaching Quality									
Question Number	Scale 1 (strongly disagree) to 5 (strongly agree)					Total	Average Response	Standard Deviation	
	1	2	3	4	5				
17	0	0	3	2	13	18	4.56	0.78	
18	0	0	3	3	12	18	4.5	0.79	
19	0	1	3	2	12	18	4.39	0.98	
Overall Rating									
Question Number	Scale 1 (strongly disagree) to 5 (strongly agree)					Total	Average Response	Standard Deviation	
	1	2	3	4	5				
21	0	1	1	4	12	18	4.5	0.86	
	Option 1*	Option 2**	Option 3***	Option 4****					
22	1	4	7	6		18	3	0.91	
	Yes	No							
24	8	10				18			
	Scale 1 (highly ineffective) to 5 (highly effective)								
	1	2	3	4	5				
25	0	0	2	5	6	13	4.31	0.75	
* it requires extensive improvements before it can be considered for use in training									
** it requires minor adjustments before it can be considered for use in training									
*** it can be used in training, but should be improved slightly									
**** it can be used in training with no improvements made									

Questions 10, 12, 13, 16, 20, 23, and 26 were qualitative and gathered participant comments which are summarized in Table 4. In general, the features of the maxSIMIO were perceived as realistic and the instructional videos hosted on the GEN platform, an online learning management system designed to teach psychomotor skills [12], were perceived as educationally useful. Comments provided suggestions for improvements, such as ensuring that maxSIMIO does not roll around when landmarking and performing the IO technique on it, making the bone harder to mimic the anatomical feeling of a human bone and reducing the thickness of the skin.

**Table 4:** Summary of the free-text comments. S# indicates participant number. Ellipses indicate illegible text.

Self-efficacy								
Question #	Comments							
10	- landmarking, equipment (S3)							
	- securing the bones a bit more (S6)							
	- stabilize the tibia in the model (S8)							
	- tighten skin - see contours better (S11)							
	- the model needs to be much more stable. The bone & skin are a bit loose and mobile (S12)							
	- do not provide one use catheters for training (S13)							
Fidelity								
Question #	Comments							
12	- no (S3)							
	- it had moved anatomically so it was difficult to landmark (S5)							
	- movement of the tibia while trying to place IO (S8)							
	- no (S9)							
	- a bit unstable? (S11)							
	- no sharps container (S13)							
13	- skin was very thick, sometimes hard to landmark (S1)							
	- It worked well, but should indicate if R or L leg (S3)							
	- practicing the anesthesia prior to if patient was conscious (S4)							
	- more secure connections along the joint/bones (S5)							
	- would be helpful to have more proximales (S7)							
	- add a fibula so med/lateral side is obvious (S10)							
	- a.....f? (S11)							
	- a bit more real (S12)							
	- the dr is always on the R of pt. You have us sitting on the abdomen (S13)							
	- the rotating tibial piece is difficult (S14)							
	- the tibia moves in the model, would be better if it didn't. Also the material is much easier to insert an IO into than real bone, can you make it harder? (S15)							
Educational Value								
Question #	Comments							
16	- R or L leg indication (S3)							
	- better material ... identify patella and tibial tuberosity (S17)							
Teaching Quality								
Question #	Comments							
20	- Good, quick videos (S3)							
	- teaching videos were great (S12)							
Overall Rating								
Question #	Comments							
23	- improve joint stability so it stays anatomically correct as it is used repeatedly							
	- add a fibula so the med/lat sides are obvious (S10)							
	- better model (S17)							
	- more anatomically specific (S18)							
	Comments							
26	- looked more anatomically correct/convincing, was more sturdy (S8)							
	- if the tibia was fixed it would be better (S15)							
	- much better (S16)							
	- the model used before is much more stable and .... more around ...? (S17)							

The clinical team also provided areas for improvement, which included “improve the joint stability, so it stays anatomically correct as it is used repeatedly”, “add a fibula so that the median/lateral sides are obvious”, and “tighten the skin [to] see contours [of the leg] better”. In addition, during the debriefing with both the development and clinical teams, other improvements highlighted were: bend in the knee, more pronounced anatomical landmarks around the knee joint, improvements to the skin attachment, and making the simulator more modular to improve the economy of longitudinal use and wear and tear effects.

**Figure 5:**
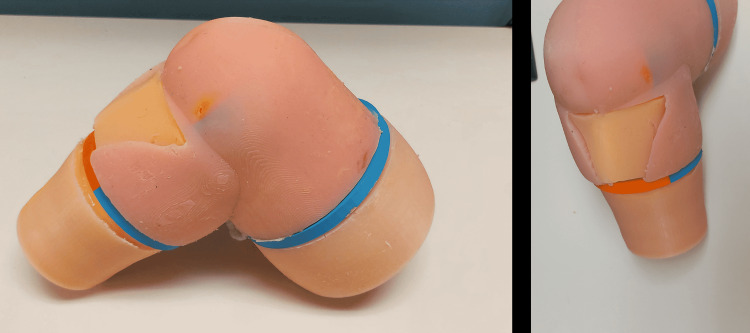
New design of the maxSIMIO based on feedback from the clinical team.

These feedback items were incorporated into a new design (Figure 5). The design is available on https://github.com/maxSIMhealth/maxSIMIOv2.0. The net costs of the re-designed simulator remained the same as the initial prototype.

## Discussion

This technical report described the development and initial end-point user feedback for the design of a 3D-printed adult proximal tibia IO simulator. This was achieved as an iterative co-design process between a development team and multiple clinical teams. The clinical team thought the simulator was a good teaching tool and provided mostly positive feedback. In terms of points for future consideration, the clinical team felt that the stability of the simulator could be improved, such as tightening the skin and securing the bones, enhancing the realism of the experience such as adding a fibula, making the bones harder, increasing the size of the patella, making the model more modular (to minimize costs related to maintenance), and improving the anatomical positioning of the knee joint (i.e., slightly bent in the knee joint). The educational value section of the assessment survey had the highest average rating (4.67/5), indicating that the clinical team believed that the intended trainees (rural and remote practitioners and trainees) will be able to gain skills through practice on the simulator. The lowest scoring question revolved around the realism of the anatomical feature of the maxSIMIO (3.88/5). The clinical team suggested stabilizing specific parts of the simulator, such as the bones inside and the entire simulator. Only eight of the 18 clinical team members had used an existing, commercially available IO simulator, and most agreed that this model was comparable except for the fact that the commercial simulators used in the past provided superior stability. Overall, the results show that the simulator allows for practice that is representative of the IO procedure to trainees with any level of experience but that it may require improvements to be a better training tool for more experienced learners. 

In comparison to existing simulators, ten tibial cartridge replacements are USD 180 from Laerdal® (Stavanger, Norway) [13] or USD 57 each from GTSimulators (Florida, USA) [14], while 3D-printing them for our simulator costs CAD 10 ($1 a replacement part). Aside from its inexpensive cost, this 3D-printed simulator has the advantage of being easily reproduced and adjusted to meet different demands (e.g., scaled to make it pediatric; altered printing parameters to create more fragile bones, etc.) in remote areas with access to a 3D printer. This characteristic of reproducibility allows rural and remote practitioners to develop and gain confidence in high acuity, low occurrence procedures before putting them into practice.

## Conclusions

In conclusion, maxSIMIO demonstrated to be a cost-effective and useful tool for training medical professionals on the IO technique. The general impressions of medical experts who used our simulator were positive, and it was highlighted that the tool allowed them to learn the rare procedure, especially those new to the skill. The clinical team provided valuable feedback to improve the simulator, such as increasing the stability and realism of the structure. We believe our simulator, after implementation of these improvements, could provide a more accessible means for rural and remote trainees and practitioners to acquire and maintain the IO technique, both in scheduled practice sessions and training sessions at home. 
